# Microsurgical management of giant malignant peripheral nerve sheath tumor of the scalp: two case reports and a literature review

**DOI:** 10.1186/1477-7819-11-269

**Published:** 2013-10-10

**Authors:** Jun Wang, Shao-wu Ou, Zong-ze Guo, Yun-jie Wang, De-guang Xing

**Affiliations:** 1 Department of Neurosurgery, the First Hospital of China Medical University, No 155 Nanjing North Street, Heping Ward, Shenyang 110001, China

**Keywords:** Malignant peripheral nerve sheath tumor, Scalp tumor, Microsurgery, Radiotherapy

## Abstract

Malignant peripheral nerve sheath tumors of the scalp are rare lesions of the nervous system. Only 14 cases have been reported to date. The field of neurosurgery has struggled with diagnosing and treating these tumors. In this report, we present two cases of giant malignant peripheral nerve sheath tumors of the scalp and retrospectively analyze the clinical features, imaging findings, pathological features, and prognoses of these two patients. Each underwent microsurgery and radiotherapy. In addition, based on a literature review, we discuss the diagnostic and therapeutic strategies used to treat these unusual lesions.

## Background

Malignant peripheral nerve sheath tumors of the scalp are rare neoplasms of the nervous system. These tumors are considered to be a subcategory of soft tissue sarcomas, because they arise from a peripheral nerve or nerve sheath cells and show divergent differentiation potentials [1-3]. A variety of terminologies, including neurofibrosarcoma, neurogenic sarcoma, malignant schwannoma, and malignant neurilemomma have been used to describe the tumor. As defined by the World Health Organization, however, the universal or standard terminology should be malignant peripheral nerve sheath tumor [1-5]. Although the incidence of malignant peripheral nerve sheath tumor of the scalp is very low, it is highly malignant and is associated with poor prognosis [1-3]. Indeed, it has always been a challenge for neurosurgeons. In this report, we present two cases that were diagnosed and treated as giant malignant peripheral nerve sheath tumor of the scalp. Both patients underwent microsurgery and radiotherapy. The pathological examinations confirmed that the resected lesions were consistent with the diagnosis of malignant peripheral nerve sheath tumor. Therefore, we retrospectively analyzed the clinical features, imaging findings, pathological features, and prognoses in each case. In addition, we reviewed the relevant literature regarding diagnostic and therapeutic strategies for treating these unusual lesions.

## Case presentation

**Figure 1:**
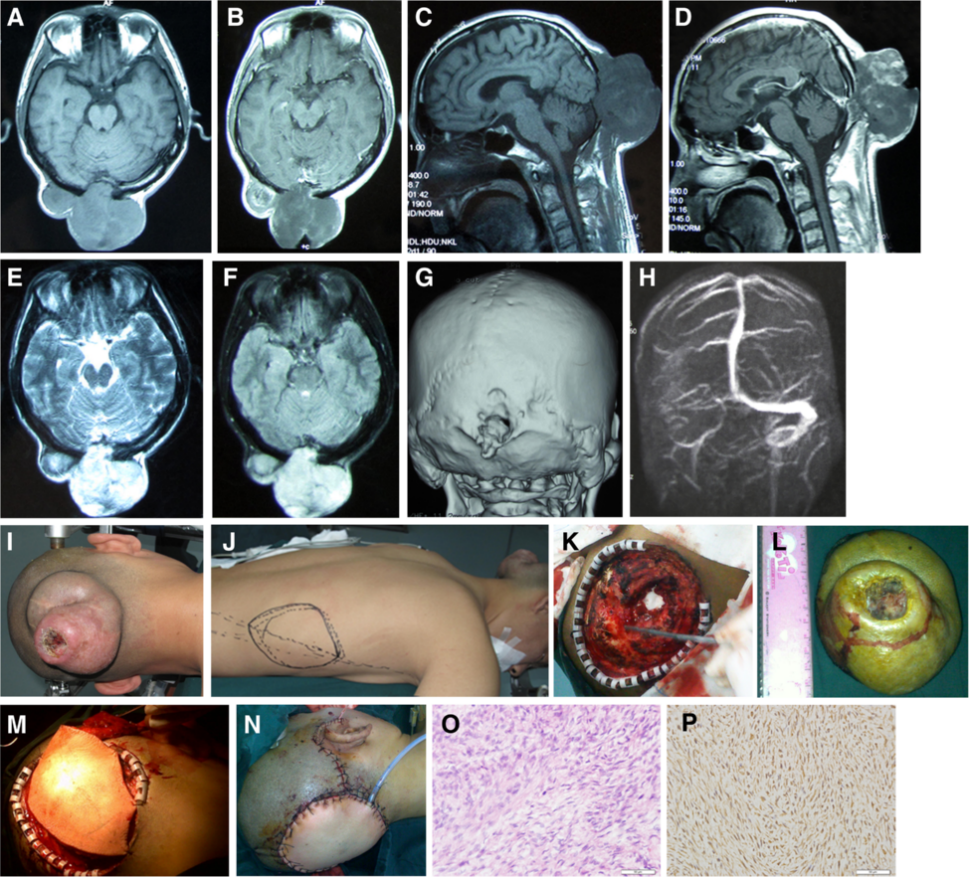
**Pre- and postoperative images of Case 1. (A) (B)** Preoperative magnetic resonance imaging (MRI) scans (cross-section) of the tumor displaying an equal T1 signal with partial enhancement and extradural extension. **(C) (D)** Preoperative MRI scans (sagittal-section) showing a partially contrast-enhancing extradural tumor in the occipital region with skull erosion. **(E) (F)** MRI fluid-attenuated inversion recovery (Flair) scan demonstrating that the lesions primarily displayed high-intensity signals. **(G)** Three-dimensional computed tomography (3-D CT) of cranium bone revealing an adjacent bone defect measuring 2.1 × 1.8 cm in the occipital area. **(H)** Magnetic resonance venography (MRV) demonstrating local compression in the right transverse sinus. **(I) (J)** Intraoperative image revealing that the middle part of the tumor was necrotic. A latissimus dorsi myocutaneous flap being planed to reconstruct the defect of the scalp. **(K) (L)** Intraoperative image demonstrating that the tumor attached to the transverse sinus was detached completely and the bone involved was also excised. The scalp defect after tumor excision measured approximately 12 × 12 cm. **(M) (N)** After large-mass excision, the scalp defect being reconstructed using a latissimus dorsi myocutaneous flap with a muscle cuff along with the vascular pedicle. The artery and vein of the flap being anastomosed with the right superficial temporal artery and vein. **(O)** Postoperative pathological examination of the tumor. Hematoxylin and eosin (H&E) staining demonstrates that the tumor cells were spindle-shaped, with variable mitotic activity and nuclear pleomorphism (×200). **(P)** Strong, positive immunoreactivity to the antibody vimentin (×200).

Case 1 was a 35-year-old male patient. The patient came to our hospital with swelling on the back of his head, which he reported had existed for 18 years. He had undergone four operations in another hospital during the past 18 years, and it had been five years since the most recent operation. The pathologic analysis was evaluated as malignant schwannoma (peripheral nerve sheath tumor) after the fourth operation. On local examination, we observed a firm, non-compressible, non-tender, non-pulsatile swelling that measured approximately 10 × 9 cm in the occipital region. The middle part of the mass was necrotic with a sickening stench (Figure 1). Generally, there were no pathologic findings on the neurologic examination, and the patient’s systemic and laboratory findings were normal. There were no clinical signs or anamnestic hints suggesting neurofibromatosis. No distant metastases were detected in computed tomography (CT) scans of the chest and abdomen. Magnetic resonance imaging (MRI) revealed a scalp swelling with bony and dural involvement measuring 10.07 × 9.38 × 6.49 cm in the occipital region, which showed nearly equal T1 and slightly long T2 signals in the lesion overall, although mixed signals were observed in various portions of the lesion. The lesion exhibited a strong signal on the fluid-attenuated inversion recovery (Flair) scan and inhomogeneous enhancement (Figure 1). Magnetic resonance venography (MRV) demonstrated local compression in the right transverse sinus. Three-dimensional computed tomography (3-D CT) of the cranium bone revealed an adjacent bone defect that measured 2.1 × 1.8 cm in the occipital area (Figure 1).

The patient underwent an occipital craniotomy and plastic and reconstructive surgery in one stage. The bone involved and a 2-cm margin of healthy tissue were excised together with the swelling. The tumor, which was attached to the dura and transverse sinus, was detached completely. Generally, the lesion was totally excised. Cranioplasty was not required because the bony defect was small (< 3 cm). After excising the large mass, the scalp defect was approximately 12 × 12 cm. Reconstruction was completed using a latissimus dorsi myocutaneous flap with a muscle cuff along with the vascular pedicle (Figure 1). The wound healed well, and no surgical complications arose. After the surgery, the diagnosis of malignant peripheral nerve sheath tumor was made through postoperative pathological examination. Light microscopy revealed that the tumor cells were spindle-shaped, with variable mitotic activity and nuclear pleomorphism (Figure 1). Focal hemorrhage and necrosis were also observed. Immunohistochemistry revealed positive immunoreactivity with vimentin (Figure 1), CD34 blood vessels, β-catenin, and ki67 (approximately 5%). The tumor was not immunoreactive with Sangtec 100 (S-100), cell keratin (CK), SMA, bcl-2, or resmin. Postoperatively, local radiotherapy was administered (60 Gy/30 d, 8 weeks after surgical excision). There was no recurrence at 20-month follow-up.

**Figure 2:**
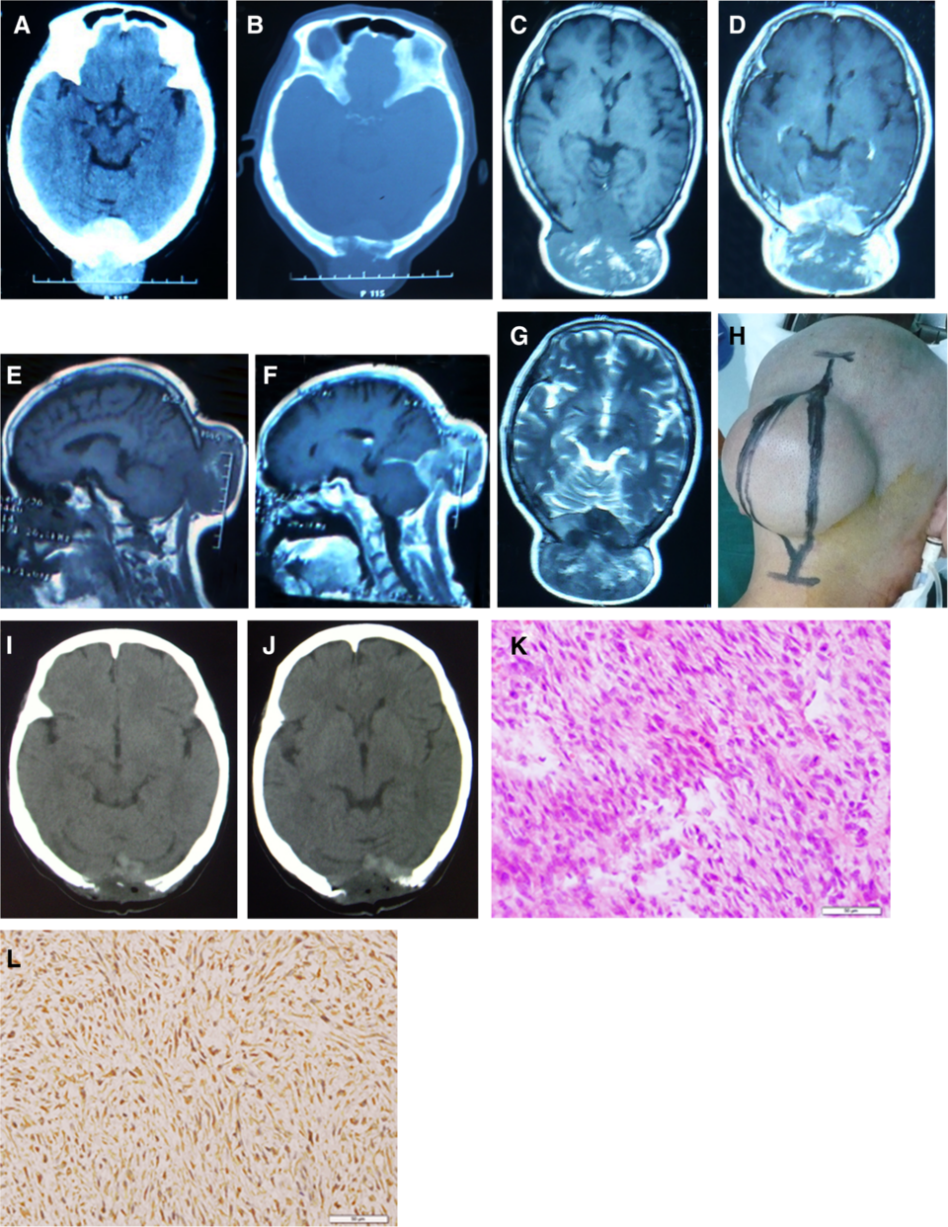
**Pre- and postoperative images of Case 2. (A) (B)** Preoperative axial computed tomography (CT) scans (one year before admission) showing a high-density lesion with partial destruction of occipital cranium. **(C) (D)** Preoperative axial magnetic resonance imaging (MRI) scans (admission) of the tumor displaying a nearly equal T1 signal (partially high signal) with peripheral enhancement and extradural extension. The swelling experienced a spurt in growth over one year. **(E) (F)** Preoperative sagittal MRI scans showing a contrast-enhancing lesion in the occipital region with dural and bony involvement. **(G)** Preoperative axial MRI scan of the tumor displaying mixed T2 signals. **(H)** The skin incision during the operation. **(I) (J)** Postoperative CT scans demonstrating that the tumor was nearly completely resected. **(K)** Postoperative pathological examination of the tumor. Hematoxylin and eosin (H&E) staining revealed that the tumor consisted of a diffuse or fascicular proliferation of spindle-shaped cells. Mitotic figures were common (×200). **(L)** Strong, positive immunoreactivity to the antibody vimentin (×200).

Case 2 was a 72-year-old female patient who presented with a swelling in the occipital region that had been gradually increasing for past four years. It had shown rapid growth during the year prior to being our hospital. Initially, the swelling was the size of a peanut. The patient had no history of trauma, fever, or infection at this site. The swelling gradually increased in size for three years and then experienced rapid growth over the next year. On examination, we found a globular (goose egg size), non-tender, non-compressible, firm to fluctuant swelling measuring approximately 10 × 10 cm in the occipital region (Figure 2). A cranial computed topography (CT) scan (one year prior to coming to our hospital) revealed a lesion that exhibited higher density to the brain parenchyma, with an adjacent bony defect measuring 4 × 4 cm in the occipital area (Figure 2). Magnetic resonance imaging (MRI) revealed a swelling with bony and dural involvement measuring 10.2 × 9. 8 × 6.7 cm in the occipital region, which showed nearly equal T1 and T2 signals. Mixed signals were also observed in various portions of the lesions, with partial enhancement in the lesion overall (Figure 2). The patient’s laboratory and systemic findings (including CT scans of the chest and abdomen) were normal. The tumor was misdiagnosed as a meningioma in the occipital area before surgery. During the surgery, the lesion was found to be firm and gray-brown in color. The intraoperative frozen sections were pathologically examined, and the tumor’s malignancy was confirmed. The lesions attached to the dura and sinus could not be detached completely because of bleeding from the transverse sinus. Still, the vast majority of the tumor was resected, and then the residual tumor was coagulated using bipolar coagulation forceps under the microscope (Figure 2).

After the surgery, the diagnosis of malignant peripheral nerve sheath tumor was confirmed through postoperative pathological examination. Light microscopy revealed that the tumor consisted of a diffuse or fascicular proliferation of long or short spindle-shaped cells, with moderately pleomorphic nuclei. Mitotic figures were common (Figure 2). Immunohistochemistry revealed positive immunoreactivity with Sangtec 100 (S-100), vimentin (Figure 2), CD34 blood vessels, CK, and ki67 (<5%). The tumor was not immunoreactive with GFAP or HMB-45. Two weeks after surgery, the patient was transferred to the Department of Radiation Oncology for local radiotherapy (59.4 Gy/33 d). In this case, the tumor recurred at nine months follow-up. The patient refused additional treatment because of her physical condition and poor prognosis. The patient was still alive at 15 months follow-up, but failed to follow up thereafter.

## Discussion

### The epidemiology of malignant peripheral nerve sheath tumor

Malignant peripheral nerve sheath tumor (MPNST) is a malignant spindle-cell tumor arising from a peripheral nerve or showing a peripheral nerve differentiation. MPNST is a rare tumor with an incidence of approximately 0.01% in the general population [2,3], with slight male predominance and accounting for 5 to 10% of all soft tissue sarcomas [1,3]. MPNST is located mainly in the trunk and extremities, such as the buttocks, thighs, brachial plexus, sciatic nerve, and paraspinal region. Primary scalp MPNST is extremely rare, with only 14 cases reported to date in English literature (Table 1) [6-18]. MPNST is considered to be associated with gene mutations, such as loss of the neurofibromatosis 1 gene protein product (neurofibroma) [13] and rearrangements of the p16 (INK4A) gene [12,14]. It has been reported that approximately one-third of MPNSTs arise *de novo*, whereas nearly 60% of all MPNSTs represent a sarcomatous degeneration of a benign neurofibroma. The remainder (approximately 10%) may be related to previous radiation at the tumor site [13,19,20].

**Table 1:** Literature review of studies of malignant peripheral nerve sheath tumor (MPNST) on the scalp

**Study**	**Sex/age**	**Tumor location**	**Neurofibromatosis type 1**	**Bone infiltration**	**Immunohistochemistry S-100**	**Treatment**	**Follow-up**
George [6]	F/56	Occipital	No	NA	+	Exc + RT	4 m, AWD
	M/50	Temporal	Yes	NA	+	Exc + RT	11 y, NED
Dabski [7]	NA/NA	Scalp	No	NA	NA	Exc	NA
Kikuchi [8]	M/59	Frontal	No	NA	+	Exc	5 y, NED
Demir [9]	M/80	Parietal	No	No	+	Exc + RT	6 m, NED
Garg [10]	M/50	Occipital	NA	Yes	+	Exc + RT	NA
Williams [11]	F/75	Scalp	No	NA	+	CT+Exc	2 y, NED
Fukushima [12]	M/38	Occipital	No	No	+	Exc	4 m, DOD
Kumar [13]	M/36	Occipital	No	Yes	+	Exc + RT	28 m, NED
Ge [14]	M/52	Parietal	Yes	Yes	+	Exc	6 m, NED
Hasturk [15]	M/44	Occipital	NA	No	+	Exc	NA
Shintaku [16]	F/59	Scalp	Yes	NA	_	Exc	18 m, DOD
Voth [17]	M/89	Parietal	No	No	+	Exc + RT	14 m, AWD
Jhawar [18]	F/43	Parietal	NA	Yes	NA	Exc	1 y, NED
Present cases	M/35	Occipital	No	Yes	_	Exc + RT	20 m, NED
	F/72	Occipital	No	Yes	+	Exc + RT	9 m (Re);
							15 m, AWD

AWD, alive with disease; CT, chemotherapy; DOD, dead of disease; Exc, excision; NA, not available; NED, no evidence of disease; Re, recurrence; RT, adjuvant radiotherapy.

Theoretically, MPNST of the scalp can originate from any site on the scalp. Of the 16 reported cases (including our patients, Table 1), however, the occipital region seemed to be the most common site, accounting for more than 50% (7 of 13, no reference in three cases). In addition, other sites, including the parietal (4 of 13), frontal (1 of 13), and temporal (1 of 13) regions were also tumor locations. Of the 16 cases (Table 1), the ratio of men to women was 10:5 (unavailable in one case), and the patients were aged 35 to 89 (mean age of 56). Three of the cases had neurofibromatosis (3 of 13, not available in three cases). Because the total number of cases of MPNST of the scalp reported to date is still small, the results concluded above need further verification.

### The clinical features of MPNST of the scalp

The initial clinical features of MPNST of the scalp are generally atypical. Most patients present with a gradually increasing swelling in the scalp (cutaneous or subcutaneous) [6-18]. On local examination, the swelling is commonly globular, firm, non-tender, non-compressible and non-pulsatile. Initially, the swelling is the size of a peanut or chestnut and then gradually increases in size. The growth of MPNST of the scalp arising *de novo*, however, occurs relatively rapidly. The tumor may invade scalp, cranium bone, and dura at the tumor site, showing superficial erosions in the scalp, destruction of the cranium bone, and involvement of the dura (extradural extension or sinus invasion). In tumors arising from sarcomatous degeneration of a benign neurofibroma, growth is relatively slow at the beginning and then becomes rapid in the last several months, suggesting malignant transformation in a relatively and previously benign tumor [13,19]. Hemorrhage and cystic degeneration in the tumor may also enlarge the lesion rapidly [15]. Symptoms of headache, vomiting, seizures, vertigo, visual impairment, or focal neurological deficits may affect patients with giant MPNST of the scalp based on the intracranial extension of the tumor.

### The imaging features of MPNST of the scalp

Although MPNSTs of the scalp generally lack specificity on imaging features at the early stage, CT or MRI scans are useful for demonstrating the relationship with surrounding structures. They may display high, equal, or low densities when the CT scans are enhanced. On MRI scans, MPNSTs of the scalp are often hypointense or isointense in T1-weighted series and hyperintense in T2-weighted and Flair sections [20]. Enhancing the lesion may be homogeneous, inhomogeneous, partial, peripheral, moderate, or dense according to the vascular densities [15,20]. Magnetic resonance venography (MRV) may be done to plan surgery for in patients with tumors located adjacent to the sinus. MRI scans often reveal features of MPNSTs that differ from those of other scalp tumors, such as lipoma and malignant melamoma. Tumors with cystic degeneration, hemorrhage, or calcification, however, may show mixed densities on CT or MRI scans, which contributes to the high chance of misdiagnosis. Of the two cases in our group (especially for Case #1, with the middle part of the tumor necrotic), MRI scans demonstrated mixed densities in T1- and T2-weighted series and inhomogeneous enhancement in contrast series. Case #2 was misdiagnosed as having malignant meningioma, and this mistake was not discovered until the surgery. We have confirmed that it is difficult to clinically diagnose MPNST of the scalp and the diagnosis of the disease relies on intraoperative and postoperative pathological findings.

### The pathological features of MPNST of the scalp

Pathological diagnosis is the gold standard for diagnosing MPNST of the scalp. Most examples of MPNST show differentiation toward Schwann cells, but MPNSTs that exhibit perineurial cells and endoneurial fibroblasts have also been reported [16]. Although MPNST is a heterogeneous group of neoplasms that shows diverse differentiation potential, routine hematoxylin-eosin (H&E) and immunohistochemical staining techniques can help diagnose the majority of MPNSTs of the scalp. H&E staining can reveal the overall morphology of the tumor cells. Under a light microscope, the cells are polygonal or spindle-shaped and exhibit signs of mitosis and nuclear pleomorphism. Focal hemorrhage and necrosis are also seen frequently [3,8,10,16,19]. Immunohistochemical staining of the tumor tissue with a variety of antibodies can diagnose the disease and differentiate it from other diseases. The S-100, EMA, Vimentin (VIM), and CD34 antibodies are highly specific to MPNST of the scalp [3,16,19,21]. For most MPNST cases, tumor cells exhibit differentiation toward Schwann cells, which is represented by immunoreactivity for S-100 protein and ultrastructurally by the presence of long cytoplasmic processes that are closely invested by a well-formed basal lamina. In some MPNST cases, tumor cells differentiate toward perineurial cells, evidenced by immunoreactivity for EMA and ultrastructurally by the presence of tight junctions, abundant pinocytotic vesicles, and an interrupted basal lamina. If tumor cells are not immunoreactive for either the S-100 protein or EMA, but are positive for vimentin, CD10, and CD34, these cells are considered to correspond to endoneurial fibroblasts [16]. Of the 16 cases (Table 1), 12 were immunoreactive for the S-100 protein (12 of 14 cases (86%); not available in two cases), suggesting a high specificity. Of the two cases whose tumor cells were not immunoreactive for the S-100 protein, one case was in our group, and his tumor cells were positive for CD34 and vimentin, suggesting the tumor is not conventional Schwann cell-derived MPNST. S-100-negative MPNSTs are uncommon. As mentioned above, perineurial cells are typically positive for EMA and negative for S-100 protein, while endoneurial fibroblasts are negative for either the S-100 protein or EMA. Other markers, such as glial fibrillary acidic protein (GFAP), cell keratin (CK) and HMB, are usually not expressed in MPNST of the scalp and are therefore used only as references. In general, a combination of antigens is used to help exclude other scalp tumors and to confirm the diagnosis of MPNST.

### Comprehensive treatment of MPNST of the scalp

The current treatment of these highly malignant tumors does not guarantee an optimistic prognosis. Surgery and local radiation therapy are currently administered to treat the disease [2-4,6-18,22,23]. Other therapies, such as chemotherapy and immunotherapy, which are usually limited to treating metastatic diseases, are still being evaluated [3,10]. The International Consensus Group has recommended that the current management of MPNST should be identical to that of any other soft tissue tumors [18]. Surgery, therefore, is the mainstay of treatment for MPNST of the scalp. The goal of surgery is to achieve complete excision of the tumor with wide (negative) margins (≥2cm) [13,17,18]. After a large mass is excised, scalp defects are common, and reconstruction always needs to be done using cutaneous or myocutaneous flaps (plastic and reconstructive surgery), such as was completed in Case #1 in our group. From a practical standpoint, the bone and dura involved should be resected together in order to avoid recurrence. When the tumor has invaded the sinus, however, total resection may be impossible due to severe bleeding in the surgery. In this case, remnants of the tumor are unavoidable, such as occurred in Case #2 in our group. Thus, as some authors have pointed out, local recurrence and disease-related deaths may be likely to be more frequent for giant MPNST of the scalp, especially in cases with high-grade tumors, incomplete surgical excision, and intracranial tumor extension [17].

As reported, surgical excision followed by adjuvant radiotherapy can improve local control [17,23]. Thus, some authors have recommended that adjuvant radiotherapy should been considered for all intermediate- and high-grade MPNST of the scalp, as well as low-grade tumors with positive margins [13,18]. We agree with the assessment that radical excision with wide margins (≥2 cm), histologic control of resection borders, and adjuvant radiation should be considered as a standard treatment for these malignant tumors [17]. Of the 16 cases (Table 1), surgical excision followed by adjuvant radiotherapy had been done in eight cases. After therapy, eight patients were (50%) alive and had no evidence of recurrence or metastasis after follow-up periods of six months to 11 years. Only two of the patients died due to metastatic disease [12,16]. It seems that the current treatment of these highly malignant tumors, however, is not that pessimistic. As some authors have pointed out, the survival rates of patients with MPNSTs were significantly better for superficial tumors, such as MPNST of the scalp [5]. Other authors, however, believe that the present data does not allow final conclusions about the prognosis of MPNST of the scalp, because these results are based on a small patient population with primarily short follow-up periods [17]. Therefore, further studies with greater numbers of patients are required to confirm the prognosis for MPNST of the scalp.

## Conclusions

In conclusion, in this paper, we have described two cases of MPNSTs of the scalp. The diagnosis of MPNST of the scalp is based on integrating clinical, imaging, histopathological, and immunohistochemical findings. Considering the high malignance and invasive growth of MPNST of the scalp, radical excision with wide margins (≥2 cm), histologic control of resection borders, and adjuvant radiation should be considered standard treatment.

## Consent

Written informed consents were obtained from the patients for publication of this Case report and any accompanying images. Copies of the written consent are available for review by the Editor-in-Chief of this journal.

## Abbreviations

CT: computed tomography; Flair: fluid-attenuated inversion recovery; H&E: hematoxylin and eosin; MPNST: malignant peripheral nerve sheath tumor; MRI: magnetic resonance imaging; MRV: magnetic resonance venography; 3-D CT: three-dimensional computed tomography.

## Competing interests

The authors declare that they have no competing interests.

## Authors’ contributions

JW wrote the initial draft. SO, ZG and YW were the neurosurgeons of the patients. DX performed the pathological examination. All authors read and approved the final manuscript.
